# Efficacy of Two Neonicotinoid Insecticides against Invasive Wood Borer *Aromia bungii* Larvae in Dietary Toxicity Test

**DOI:** 10.3390/insects12070592

**Published:** 2021-06-29

**Authors:** Eiriki Sunamura, Shigeaki Tamura, Hisatomo Taki, Hiroki Sato, Etsuko Shoda-Kagaya, Tadahisa Urano

**Affiliations:** 1 Forestry and Forest Products Research Institute, Matsunosato 1, Tsukuba, Ibaraki 305-8687, Japan; tamus@affrc.go.jp (S.T.); htaki@affrc.go.jp (H.T.); hirokis@affrc.go.jp (H.S.); eteshoda@affrc.go.jp (E.S.-K.); 2 Kansai Research Center, Forestry and Forest Products Research Institute, Nagaikyutaroh 68, Momoyama-cho, Fushimi-ku, Kyoto 612-0855, Japan; urano@ffpri.affrc.go.jp

**Keywords:** *Aromia bungii*, biological invasions, Cerambycidae, chemical control, dinotefuran, neonicotinoids, pest management, thiamethoxam, wood boring insects

## Abstract

**Simple Summary:**

This study investigated the effects of two neonicotinoid insecticides (thiamethoxam and dinotefuran) on alien wood borer *Aromia bungii*, which invaded Japan recently. Small neonates and large larvae were fed artificial diet with different insecticide concentrations and then reared for 3 (neonates) or 12 (large larvae) weeks in the laboratory. Diet excavation immediately dropped in larvae exposed to high concentrations of both insecticides (≥1 ppm in neonates and ≥10 ppm in large larvae). Their growth was significantly suppressed, and the survival rate gradually declined over time (≥87% decline over 12 weeks in large larvae). These effects were similar between neonates and large larvae, but neonates were affected more by lower insecticide concentrations than large larvae. The two insecticides gradually debilitate *A. bungii* larvae. In practical use, rapid suppression of *A. bungii* wood boring damage can be expected by injecting these insecticides into infested trees. However, a relatively long–term retention of the insecticides may be required to kill the larvae in the trees, especially large larvae. Neonates may be controlled with less insecticides and shorter exposure than large larvae.

**Abstract:**

In recent years, insecticide trunk injection was put into practical use for controlling wood boring pests. However, few studies have investigated the dose–response relationships between insecticides and wood–boring pests in detail. This study used two commercial formulations of the neonicotinoid insecticides thiamethoxam and dinotefuran and investigated their dose–response relationships with invasive wood borer *Aromia bungii* (Coleoptera: Cerambycidae) larvae. Neonates and late instar larvae were reared with an artificial diet containing different insecticide concentrations (0.01–100 ppm) in the laboratory, and their diet excavation activity, survival rate, and weight change were recorded. Diet excavation immediately dropped in larvae exposed to high concentrations of thiamethoxam or dinotefuran (≥1 ppm in neonates and ≥10 ppm in late instar larvae). The weight and survival rate gradually declined over 12 weeks in late instar larvae. These results suggest that the two neonicotinoids intoxicate and debilitate *A. bungii* larvae gradually to death. In practical use, rapid suppression of *A. bungii* wood boring damage can be expected by trunk injection of neonicotinoid insecticides. However, a relatively long-term retention of the insecticides may be required to kill large larvae. Neonates may be controlled with lower insecticide dosage and shorter exposure than larger larvae.

## 1. Introduction

Wood boring pests can cause serious damage to tree stands. Many of these pests spend most of their lives at the larval stage inside trees; thus, efficient control of larvae is important to prevent damage. However, control of larvae is not easy due to their lack of visibility from the tree surface and technical difficulty in delivering control agents [1]. These difficulties have allowed the exotic Asian longhorn beetle *Anoplophora glabripennis* (Coleoptera: Cerambycidae), its close relative *An. chinensis*, and the emerald ash borer *Agrilus planipennis* (Coleoptera: Buprestidae) to kill millions of forest and urban trees in North America and Europe since the late 1990s [2,3]. Traditionally, insertion of aerosol insecticides into frass holes has been used as a control measure for longhorn beetle larvae [4]. However, this is not a practical solution for wood–boring pests, because treatment of individual frass holes is labor-intensive when the pest density is high, larval feeding tunnels are often intricate and the insecticides do not reach the larvae, and frass holes at high positions are out of reach [5].

Trunk injection of insecticides is a new, alternative method to control wood–boring pests inside trees. In this method, systemic insecticides are applied to tree trunks and distributed throughout the trees via xylem water uptake [6,7]. Soil drenching is a similar method that exploits xylem water uptake to deliver insecticides within trees. Trunk injection is most frequently used to control foliage and phloem feeding pests in an urban environment because this method does not cause insecticide drift, which is a major concern in insecticide spraying [8,9,10,11]. Some formulations have been commercialized for wood–boring pests [3,4]. These include synthetic neonicotinoids, organophosphates, macrolides, and natural azadirachtin as active ingredients and intoxicate wood–boring pests primarily via oral ingestion of insecticide–permeated wood tissues.

However, the efficacy of insecticide trunk injection (or soil drenching) against wood–boring pests has been reported by only a few field experiments [12,13,14,15,16]. Furthermore, detailed studies on the dose–response relationships between insecticides and wood–boring pests are scarce [17,18]. Such knowledge is important for improving dose setting and efficacy evaluation in practical use. Previous studies have suggested that neonicotinoid and macrolide insecticides show strong antifeedant effects against *An. glabripennis*, *Plectrodera scalator* (related species), and *Ag. planipennis* larvae and require relatively long-term exposure to kill those larvae [17,18]. There is value in further verifying whether these effects are found among other wood–boring pests, comparing the dose–response relationships between larvae at different developmental stages, and investigating whether larvae can develop normally after temporary exposure to the insecticides.

The red–necked longhorn beetle *Aromia bungii* (Coleoptera: Cerambycidae) is an invasive wood–boring pest [5,19]. This species was originally distributed in China, the Korean peninsula, and surrounding areas but has now invaded Germany, Italy, and Japan in the past decade. The larvae infest Amygdaloideae (Rosaceae) trees such as cherries, plums, and peaches. They feed on the inner bark, cambium, and outer xylem. When individual trees are infested by a large number of larvae, they get damaged, wilt, and die [20]. In 2019, two commercial insecticides containing neonicotinoid compounds thiamethoxam or dinotefuran as the active ingredient became available for trunk injection against *Ar. bungii* larvae in cherry blossom trees in Japan [15,21]. Trunk injection of these insecticides effectively stops frass ejection from *Ar. bungii* infested trees [15,21]. *Aromia bungii* adults do not feed on foliage and twigs [5]; thus, trunk-injected insecticides do not reach them and target only larvae [21].

In this study, we evaluated the efficacy of the two neonicotinoid insecticides against *Ar. bungii* larvae in a laboratory dietary toxicity test. We used larvae at two different developmental stages, neonates and late instar, and fed them an artificial diet with different concentrations of insecticides. Neonates and late instar larvae were reared for 3 and 12 weeks, respectively. Their diet excavation activity, survival rate, and weight change were recorded. We also moved some larvae that survived the dietary toxicity test to an intact diet and investigated whether they recovered from growth suppression.

## 2. Materials and Methods

### 2.1. Insects and Insecticides

*Aromia bungii* female adults were collected from some distribution areas in the Kanto region, Japan (e.g., Soka City [35°84′ N, 139°83′ E] and Sano City [36°28′ N, 139°55′ E]). The female adults were kept in plastic cups and allowed to lay eggs on filter paper. Hatched neonates (mean ± standard deviation (SD) weight per individual = 0.30 ± 0.016 mg) were either used for a dietary toxicity test within 2 days after hatch (August 2020) or reared until the late instar stage (mean ± SD weight per individual = 1055 ± 207 mg) (November 2020). Late instar larvae were chosen by weight. The number of times the larvae molted was not counted during rearing, and the exact instar number of larvae was unknown.

An artificial diet slightly modified from Urano (2021) was used to rear the larvae [22]. Ingredients were 34% Silkmate PM (Nosan Corporation, Kanagawa, Japan), 6% dried yeast Ebios (Asahi Group Foods, Ltd., Tokyo, Japan), 10% cherry wood dust, and 50% distilled water. This diet also served as a control in the dietary toxicity test.

Two trunk injection insecticides registered for controlling *Ar. bungii* larvae in Japan were used, 4% thiamethoxam solution (ATTRAC; Syngenta Japan Co., Ltd., Tokyo, Japan; 96% consists of organic solvent, surfactant, etc.) and 8% dinotefuran solution (Wood–Star; Sankei Chemical Co., Ltd., Kagoshima, Japan; 92% consists of water, organic solvent, etc.).

### 2.2. Dietary Toxicity Test against Neonates

A toxic diet was prepared by diluting the insecticides with distilled water and replacing the 50% distilled water portion in the artificial diet with the insecticide dilution. The concentration of thiamethoxam and dinotefuran in the diet was adjusted to 0.01, 0.1, 1, and 10 ppm. In the field, the two insecticides are expected to reach these concentrations in cherry tree sapwood by injecting the registered dosages [23]. The prepared diets (ca. 20 g) were packed firmly into individual glass Petri dishes (60 mm diameter × 15 mm height). Ten replicate dishes were made for each concentration. Ten small holes (ca. 1 mm diameter × 2 mm depth) were prepared in each diet with forceps. Individual holes were ca. 10 mm apart. One neonate was introduced into each hole (10 neonates per dish, 100 neonates per concentration). The dishes were kept in the dark at 25 °C.

The larval boring rate, the survival rate, and larval weight were measured. The larval boring rate was investigated at days 1, 2, 3, 7, 14, and 21 after introduction. Larvae ejecting frass from introduction holes and being no longer visible were regarded as bored. The survival rate was measured after 21 days by stopping the diet and checking for live and dead larvae inside. The larval weight was measured at days 0 and 21. Because neonates were small, the total weight of live neonates was measured for each dish.

At day 21, 60 larvae that survived 1 ppm dinotefuran treatment were moved to an insecticide-free artificial diet. The diet (ca. 100 g) was packed firmly into individual glass Petri dishes (100 mm diameter × 15 mm height), and 30 larvae were introduced into each dish (2 dishes in total). The dishes were kept in the dark at 25 °C. The number of surviving individuals and their weights were recorded 6 weeks (42 days) later.

### 2.3. Dietary Toxicity Test against Late Instar Larvae

A toxic diet containing 0.01, 0.1, 1, 10, and 100 ppm of thiamethoxam or dinotefuran was prepared, as previously described. The prepared diet (ca. 20 g) was packed firmly into individual glass Petri dishes (60 mm diameter × 15 mm height). Fifteen replicate dishes were made for each concentration. A dent (ca. 50 mm length × 10 mm width × 10 mm depth) was created in the center of the diet, and one larva was introduced into the dent (15 larvae per concentration). The larvae were weighed individually just before introduction. At each insecticide concentration, the larval weight was not different from the control (generalized linear models (GLMs), −0.62 < *t* < 0.035, *p* > 0.05). The dishes with late instar larvae were kept in the dark at 25 °C. The diet was replaced with a fresh one every 2 weeks.

**Figure 1:**
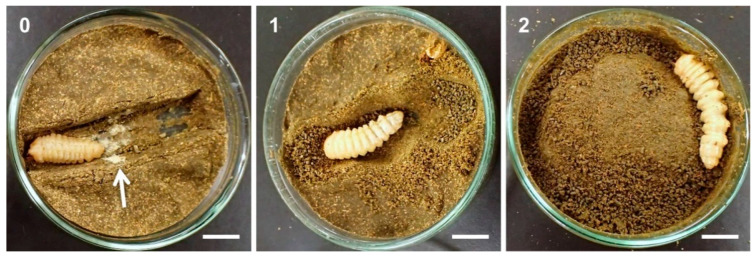
Diet excavation by *Aromia bungii* late instar larvae. Excavation score: 0 = no trace of excavation, 1 = moderate excavation (central dent not fully filled), and 2 = vigorous excavation (central dent fully filled and tunnel extending to other parts). The arrow in score 0 image shows abnormal white, muddy excretion by the larva, while a lot of normal granular feces can be found in the score 1 and 2 images. Scale bars = 1 cm.

For each larva, survival, weight, level of diet excavation, and property of excretion were investigated 2, 4, 6, 8, 10, and approximately 12 weeks (14, 28, 42, 56, 70, and 83 days) after introduction. The level of diet excavation was scored as follows: 0 = no trace of excavation, 1 = moderate excavation (central dent not fully filled with excretion), and 2 = vigorous excavation (central dent fully filled and tunnel extending to other parts) (Figure 1). Regarding the property of excretion, the presence or absence of white, muddy excretion was recorded. This is different from the normal green, granular excretion of larvae (green was the color of the artificial diet) (Figure 1). Cerambycid larvae of tribe Cerambycini and Callichromatini (Cerambycinae), including *Ar. bungii*, discharge white, calcium carbonate-rich excretion, and use it to close their pupal chambers [5]. We investigated whether the white muddy excretion by *Ar. bungii* larvae included calcium carbonate by soaking five randomly chosen samples in 10% citric acid. If calcium carbonate was included, the samples should foam and dissolve.

After 12 weeks, four larvae that survived insecticide exposure (one larva, 10 ppm thiamethoxam treatment; two larvae, 10 ppm dinotefuran treatment; and one larva, 100 ppm dinotefuran treatment) were moved to individual glass Petri dishes (60 mm diameter × 15 mm height) with an insecticide-free artificial diet (ca. 20 g). The dishes were kept in the dark at 25 °C. The diet was replaced with a fresh one every 2 weeks. The larvae’s survival and weight were measured 6 weeks (42 days) later.

### 2.4. Statistical Analysis

To examine the effects of insecticides on neonate boring into the diet, generalized linear mixed models (GLMMs) were constructed with treatment (insecticide concentration) and time (days elapsed) as fixed factors, the test dish as a random factor, and the larval diet–boring rate as the response variable. For each insecticide, the effect of treatment and interaction between treatment and time were analyzed. To test the killing efficacy of each insecticide against neonates, GLMs were constructed with treatment (insecticide concentration) as a fixed factor and survival rate as the response variable. To evaluate the effects of insecticides on neonate growth, GLMMs were constructed with treatment (insecticide concentration) and time (days elapsed) as fixed factors, the test dish as a random factor, and the mean larval weight per dish as the response variable. For each insecticide, the effect of treatment and interaction between treatment and time were analyzed. In the GLMs and GLMMs, the response variables were assumed to follow Gaussian distribution.

To examine the killing efficacy of each insecticide against late instar larvae, GLMs were constructed with treatment (insecticide concentration) and time (weeks elapsed) as fixed factors and the biweekly survival rate (rate of survival for 2 weeks after the last observation) as the response variable. The effects of treatment and interaction between treatment and time were analyzed. The 50% lethal concentration (LC_50_) and 50% lethal time (LT_50_) were calculated by probit analyses. To examine the effects of insecticides on the growth of late instar larvae, GLMMs were constructed with treatment (insecticide concentration) and time (weeks elapsed) as fixed factors, the individual larva as a random factor, and the biweekly growth rate (relative weight of larva compared to the last measurement) as the response variable. For each insecticide, the effects of treatment and interaction between treatment and time were analyzed. To evaluate the effects of insecticides on diet excavation, GLMMs were constructed with treatment (insecticide concentration) and time (weeks elapsed) as fixed factors, the individual larva as a random factor, and the diet excavation score as the response variable. For each insecticide, the effects of treatment and interaction between treatment and time were analyzed. In the GLMs and GLMMs, the response variables were assumed to follow Gaussian distribution.

All statistical analyses were performed using R version 3.6.0 [24], and *p* < 0.05 was considered statistically significant.

## 3. Results

### 3.1. Dietary Toxicity Test against Neonates

**Figure 2:**
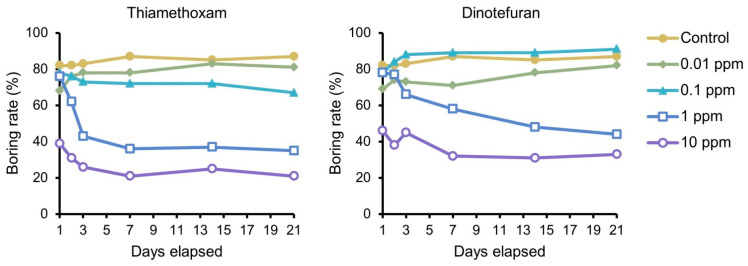
*Aromia bungii* neonates’ diet–boring rate in the dietary toxicity test.

The neonate boring rate was initially similar in the 0.1 and 1 ppm thiamethoxam treatment and control groups at day 1 (Figure 2). However, some larvae in these treatment groups later stopped boring and returned to the diet surface; hence, the boring rate declined. The rate was low from the start in the 10 ppm thiamethoxam group. In the 1 and 10 ppm dinotefuran groups, the boring pattern was similar to that in the 1 and 10 ppm thiamethoxam groups, respectively (Figure 2). A treatment–time interaction was detected in the 0.1, 1, and 10 ppm thiamethoxam groups as well as in the 1 and 10 ppm dinotefuran groups (Appendix A).

**Figure 3:**
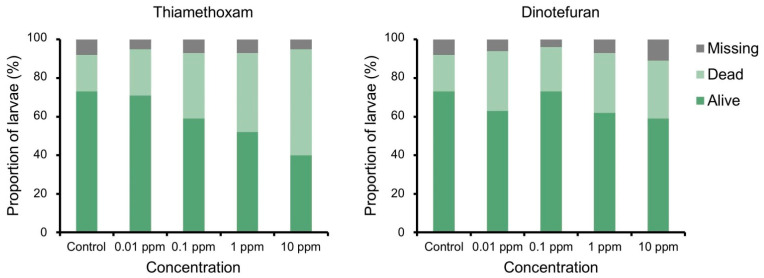
State of *Aromia bungii* neonates after 3 weeks’ exposure to insecticides.

The survival rate (52% ± 15% and 40% ± 12%, respectively) was lower in the 1 and 10 ppm thiamethoxam groups compared with the control group (73% ± 19%) (*t* = −2.8, *p* < 0.01 and t = −4.5, *p* < 0.001, respectively) (Figure 3). In other treatment groups, the survival rate did not differ from that of the control group. Despite a careful search, some neonates (≤11%) could not be detected at day 21.

The weight increased by ≥14 times during 21 days in the control, 0.01 and 0.1 ppm thiamethoxam, and 0.01 and 0.1 ppm dinotefuran groups (Table 1). At higher concentrations of thiamethoxam and dinotefuran, the weight stayed within a similar range or even decreased. A negative treatment–time interaction was detected in the 0.01, 0.1, 1, and 10 ppm thiamethoxam groups as well as the 1 and 10 ppm dinotefuran groups (Appendix B). The diet–boring rate, survival rate, and weight measures for individual test dishes are presented in Spreadsheet S1.

In addition, 5 of 60 neonates remained alive at 6 weeks after replacing the dinotefuran 1 ppm diet with an insecticide-free diet. The individual weight increased from ca. 0.3 to 158, 142, 97, 84, and 58 mg, respectively. The remaining 55 individuals were either dead or missing.

### 3.2. Dietary Toxicity Test against Late Instar Larvae

**Figure 4:**
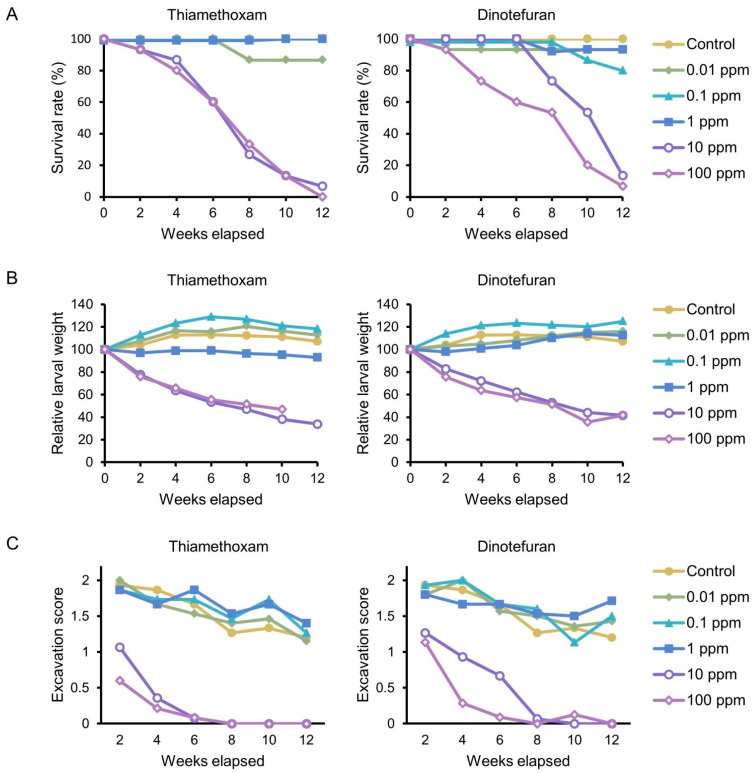
Results of dietary toxicity test against *Aromia bungii* late instar larvae. (**A**) Survival rate. (**B**) Weight change. The relative weight is shown with an initial individual weight as 100. (**C**) Diet excavation. See the explanation for excavation scores in Figure 1.

The survival rate of late instar larvae was ≥80% throughout 12 weeks at ≤1 ppm of thiamethoxam and dinotefuran (Figure 4A). In contrast, the survival rate gradually declined to ≤13% at ≥10 ppm of thiamethoxam and dinotefuran. A negative treatment–time interaction was detected at ≥10 ppm of thiamethoxam and dinotefuran (Appendix C).

LC_50_ at 12 weeks was 2.2 ppm for thiamethoxam (95% confidence interval (CI): 0.88–5.73) and 2.2 ppm for dinotefuran (95% CI: 0.78–6.51). LT_50_ was 47.1 days for 10 ppm of thiamethoxam (95% CI: 40.0–54.1), 45.8 days for 100 ppm of thiamethoxam (95% CI: 38.9–52.6), 68.6 days for 10 ppm of dinotefuran (95% CI: 63.1–75.0), and 49.8 days for 100 ppm of dinotefuran (95% CI: 41.5–58.1).

The weight gradually increased during the first 4–6 weeks in the 0.01 and 0.1 ppm thiamethoxam, as well as control groups (Figure 4B). The weight then became flat or slightly decreased because some individuals became fully grown. Full-grown larvae can be distinguished from immature larvae based on their lighter coloration in the mouth parts and prothorax [25]. In the 1 ppm thiamethoxam group, the weight remained at the same level through 12 weeks. In the 10 and 100 ppm thiamethoxam groups, the weight continued to decrease and reached <50% of the initial weight at 12 weeks. Negative effects of treatment on larval growth were detected in 1, 10, and 100 ppm thiamethoxam groups (Appendix D). In the 100 ppm thiamethoxam group, a positive treatment–time interaction was detected due to survival of individuals whose weight loss was milder. The weight declined to <50% in the 10 and 100 ppm dinotefuran groups (Figure 4B), and a negative effect of treatment on larval growth was detected (Appendix D). In the 100 ppm dinotefuran group, a positive treatment–time interaction was detected. In the 0.01 and 1 ppm dinotefuran groups, the weight increased more slowly compared with the control group (Figure 4B). At these concentrations, positive treatment–time interactions were detected (Appendix D) due to delayed growth.

Diet excavation by larvae was vigorous in the control group and at low insecticide concentrations (0.01–1 ppm) (Figure 4C). The excavation level declined in full-grown larvae, but most larvae showed some trace of excavation, even after becoming full-grown. The excavation level was much lower at higher insecticide concentrations (10 and 100 ppm). The larvae initially showed moderate excavation, but the activity dropped to nearly zero within 4–8 weeks. We detected a positive treatment–time interaction in relation to the excavation score in the 1 ppm thiamethoxam group (Appendix E), a negative interaction in the 10 ppm thiamethoxam group, and a negative effect of treatment in the 100 ppm thiamethoxam group. Similar interactions and effects were also detected in the dinotefuran groups.

White muddy excretion was observed in ≥80% of larvae fed with high concentrations of insecticides (10 and 100 ppm) (Figure 1 and Table 2). In many cases, larvae repeatedly excreted abnormal feces until death. However, abnormal excretion was rarely observed at lower concentrations of insecticides and in the control group. When white excretion samples were soaked in 10% citric acid, neither foaming nor dissolution was observed. The mortality, weight, diet excavation score, and abnormal excretion records for individual larvae are presented in Spreadsheet S2.

One of four late instar larvae remained alive at 6 weeks after replacing the toxic diet (10 ppm dinotefuran) with the intact diet. The weight of this individual was 1148 mg at the beginning of the dietary toxicity test, declined to 549 mg with 10 ppm dinotefuran treatment, and then recovered to 706 mg with the insecticide-free diet. The other three late instar larvae died within two weeks despite replacing the toxic diet with the intact diet (one individual from the 10 ppm thiamethoxam group, one from the 10 ppm dinotefuran group, and one from the 100 ppm dinotefuran group).

## 4. Discussion

When high concentrations of thiamethoxam or dinotefuran were included in the diet, the diet excavation rapidly declined in both *Ar. bungii* neonates and late instar larvae. Their growth was suppressed, and their weight significantly declined at the late instar stage. The larvae gradually died over several weeks. These results suggest that the two neonicotinoids intoxicate and debilitate *Ar. bungii* larvae gradually to death. At low concentrations, these insecticides have little impact on larval diet excavation or survival, but larval growth is suppressed at such sublethal concentrations. For instance, the weight of late instar larvae did not change through 12 weeks after 1 ppm thiamethoxam treatment but increased with 0.01 and 1 ppm dinotefuran treatment, although more slowly than the control group.

*Aromia bungii* neonates may be ca. 10 times more susceptible to thiamethoxam and dinotefuran than late instar larvae, based on the growth suppression level. In addition, neonates require a relatively short exposure to be killed (≤3 weeks at 1 ppm of dinotefuran). In the field, trunk injection of a relatively low dose of neonicotinoids in the *Ar. bungii* reproductive season may eliminate hatched neonates and effectively prevent damage to trees. Previous laboratory and field evaluations of insecticides against wood–boring pest larvae have focused mainly on large larvae. Our study indicates that neonates’ insecticide susceptibility evaluation may provide useful information for practical control.

The antifeedant effect of neonicotinoids has been found in some insect species [26,27,28,29,30], including a few wood–boring pest larvae [17,18]. Moreover, previous studies required a relatively long period to kill larvae using neonicotinoids [17,18], which is consistent with our study. In Cerambycidae, the LC_50_ of a neonicotinoid compound imidacloprid (14 weeks’ exposure) was 4.92 ppm for *An. glabripennis* (mean ± SD larval weight: 1.23 ± 0.09 g) and 1.78 ppm for *P. scalator* (mean ± SD larval weight: 0.82 ± 0.02 g) [17]. The LT_50_ of 16 ppm imidacloprid was 14.0 weeks for *An. glabripennis* and 9.26 weeks for *P. scalator*. At 160 ppm, the antifeedant effect of imidacloprid suppressed larval intake of the compound and LT_50_ was further prolonged. In our study, different varieties of neonicotinoid, thiamethoxam and dinotefuran, were used. The weight and LC_50_ of *Ar. bungii* late instar larvae were similar to those beetles, suggesting similar insecticidal potency among imidacloprid, thiamethoxam, and dinotefuran. Meanwhile, LT_50_ was substantially shorter and was not extended by increasing the insecticide concentration from 10 to 100 ppm. This suggests that thiamethoxam and dinotefuran have less antifeedant effect than imidacloprid or that *Ar. bungii* larvae are less susceptible to the antifeedant effect of neonicotinoids than other species. Previous studies have suggested that thiamethoxam and clothianidin have less antifeedant effect than imidacloprid in other insects [30,31].

To the best of our knowledge, abnormal excretion similar to that observed in our study has not been reported in other insects exposed to neonicotinoids. A lack of chemical reaction between excretion and citric acid indicates the absence of calcium carbonate in the excretion; thus, it was not excreted to construct the pupal chamber but was likely a symptom of intoxication. Other than abnormal feces, we observed regurgitation of a transparent liquid from the larval mouth once. Regurgitation was also reported in *Ag. planipennis* adults that ingested dinotefuran [14]. *Aromia bungii* larvae exposed to thiamethoxam and dinotefuran may have lost weight by abnormal excretion and regurgitation.

Intoxication and starvation can be possible causes of the death of *Ar. bungii* larvae exposed to thiamethoxam and dinotefuran because these compounds may possess both killing and antifeedant effects. The relative contribution of intoxication and starvation to larval weakening and death is unknown, but our late instar larvae test provided supporting evidence that intoxication plays a specific role. First, larvae exposed to the two insecticides initially showed moderate diet excavation activity, which declined over time. This suggests that the larvae initially fed on the toxic diet, became intoxicated, and gradually weakened. Second, abnormal excretion continued until death. This suggests that the larvae were continuously affected by the insecticides. Third, the mortality of *Ar. bungii* larvae was higher than that of *An. glabripennis* larvae from the previous study where the antifeedant effect was prominent, as described before [17]. In addition, the weight decrease in *Ar. bungii* larvae was more obvious that that in *An. glabripennis* larvae. These effects are likely from intoxication rather than starvation.

The rapid suppression of larval diet excavation by thiamethoxam and dinotefuran suggests that *Ar. bungii* wood–boring damage can be quickly suppressed by trunk injection of these two insecticides in the field. Accordingly, frass ejection can be expected to decrease or quickly stop. In fact, trunk injection of thiamethoxam and dinotefuran formulations stops a majority of frass ejection from *Ar. bungii*-infested trees within a few weeks [15,21].

Our results also suggest that there is a time lag between the stopping of frass ejection and the death of *Ar. bungii* larvae in the field. Based on our calculations, a persistence of 0.8–6.5 ppm (95% CI of LC_50_) insecticides for several weeks might be required to kill large larvae within trees. If the insecticide concentration declines early to an insufficient level, some larvae may resume feeding and growing, as shown by our tests. However, a precise exposure period required to kill larvae could not be verified in this study. Most of the neonates exposed to 1 ppm of dinotefuran for three weeks were alive, but most of them then died during the next six weeks of isolation from the insecticide. This suggests that the neonates ingested a lethal amount of dinotefuran within the first three weeks. Late instar larvae also died during the recovery test. Further research is needed to clarify the desirable insecticide residue level and duration to control *Ar. bungii* larvae in trees.

## 5. Conclusions

Two neonicotinoids thiamethoxam and dinotefuran gradually intoxicate and debilitate *Ar. bungii* larvae and show some antifeedant effect, leading to rapid suppression of larval feeding activity. To kill the larvae, a longer exposure period may be required. Neonates may be controlled with a lower insecticide dosage and shorter exposure than larger larvae.

## Figures and Tables

**Table 1:** Weight change of *Aromia bungii* neonates in the dietary toxicity test.

Insecticide	Active Ingredient Concentration	Mean ± SD Weight of Individual Larva (mg)		Growth Rate (21 Days/0 Day)
		0 Day	21 Days	
Control (water)	0 ppm	0.30 ± 0.01	8.36 ± 2.50	28
Thiamethoxam	0.01 ppm	0.32 ± 0.02	5.35 ± 2.04	17
	0.1 ppm	0.30 ± 0.01	4.13 ± 1.96	14
	1 ppm	0.30 ± 0.01	0.26 ± 0.02	0.87
	10 ppm	0.31 ± 0.01	0.23 ± 0.04	0.74
Dinotefuran	0.01 ppm	0.31 ± 0.01	6.12 ± 2.96	20
	0.1 ppm	0.31 ± 0.01	8.25 ± 4.28	27
	1 ppm	0.30 ± 0.01	0.34 ± 0.05	1.1
	10 ppm	0.30 ± 0.02	0.23 ± 0.02	0.77

**Table 2:** Abnormal excretion by *Aromia bungii* late instar larvae in the dietary toxicity test.

Insecticide	Active Ingredient Concentration	Rate of Individuals Excreted (%)		Biweekly Rate of Individuals Excreted (%)					
		≥Once	≥Twice	2W	4W	6W	8W	10W	12W
Control (water)	0 ppm	0	0	0	0	0	0	0	0
Thiamethoxam	0.01 ppm	0	0	0	0	0	0	0	0
	0.1 ppm	0	0	0	0	0	0	0	0
	1 ppm	7	0	0	0	0	0	0	7
	10 ppm	93	67	33	29	69	78	75	50
	100 ppm	87	47	20	29	42	56	60	0
Dinotefuran	0.01 ppm	0	0	0	0	0	0	0	0
	0.1 ppm	7	0	0	0	0	7	0	0
	1 ppm	0	0	0	0	0	0	0	0
	10 ppm	87	53	0	0	7	60	82	38
	100 ppm	80	47	13	43	27	44	63	67

## Data Availability

All data analyzed in this study are included in this article and Supplementary Materials.

## Supplementary Materials

The following are available online at https://www.mdpi.com/article/10.3390/insects12070592/s1, Spreadsheet S1: Neonates’ diet–boring rate, survival rate, and weight; Spreadsheet S2: Late instar larvae’s mortality, weight, diet excavation score, and record of abnormal excretion.

## Appendix A

**Table A1:** Parameter estimates for GLMMs to explain the diet–boring rate by *Aromia bungii* neonates.

Insecticide	Effect	Estimate	SE	*t*	*p*
Thiamethoxam	0.01 ppm	−8.97	5.91	−1.52	0.13
	0.1 ppm	−6.04	5.91	−1.02	0.31
	1 ppm	−22.2	5.91	−3.76	<0.001
	10 ppm	−50.6	5.91	−8.56	<0.001
	Day ^1^	0.23	0.20	1.16	0.25
	0.01 ppm:day ^2^	0.25	0.28	0.88	0.38
	0.1 ppm:day	−0.66	0.28	−2.35	<0.05
	1 ppm:day	−1.74	0.28	−6.18	<0.001
	10 ppm:day	−0.82	0.28	−2.92	<0.005
Dinotefuran	0.01 ppm	−12.3	5.60	−2.20	<0.05
	0.1 ppm	0.52	5.60	0.093	0.93
	1 ppm	−7.08	5.60	−1.27	0.21
	10 ppm	−40.1	5.60	−7.17	<0.001
	Day	0.23	0.17	1.36	0.18
	0.01 ppm:day	0.31	0.24	1.29	0.20
	0.1 ppm:day	0.21	0.24	0.85	0.39
	1 ppm:day	−1.93	0.24	−8.00	<0.001
	10 ppm:day	−0.84	0.24	−3.48	<0.001

^1^ Days elapsed after start of insecticide exposure (1, 2, 3, 7, 14, and 21 days). ^2^ Interaction between *X* ppm treatment and elapsed days.

## Appendix B

**Table A2:** Parameter estimates for GLMMs to explain the growth rate of *Aromia bungii* neonates.

Insecticide	Effect	Estimate	SE	*t*	*p*
Thiamethoxam	0.01 ppm	0.017	0.53	0.033	0.97
	0.1 ppm	0.0070	0.53	0.013	0.99
	1 ppm	0.0040	0.53	0.007	0.99
	10 ppm	0.014	0.53	0.025	0.98
	Day ^1^	0.38	0.025	15	<0.001
	0.01 ppm:day ^2^	−0.14	0.036	−4.0	<0.001
	0.1 ppm:day	−0.20	0.036	−5.6	<0.001
	1 ppm:day	−0.39	0.036	−11	<0.001
	10 ppm:day	−0.39	0.036	−11	<0.001
Dinotefuran	0.01 ppm	0.0080	0.82	0.010	0.99
	0.1 ppm	0.0089	0.82	0.011	0.99
	1 ppm	0.0014	0.82	0.002	0.999
	10 ppm	−0.0020	0.82	−0.002	0.998
	Day	0.38	0.039	9.9	<0.001
	0.01 ppm:day	−0.11	0.055	−1.9	0.055
	0.1 ppm:day	−0.0030	0.055	−0.10	0.92
	1 ppm:day	−0.38	0.055	−7.0	<0.001
	10 ppm:day	−0.39	0.055	−7.0	<0.001

^1^ Days elapsed after start of insecticide exposure (0 and 21 days). ^2^ Interaction between *X* ppm treatment and elapsed days.

## Appendix C

**Table A3:** Parameter estimates for GLMs to explain the survival rate of *Aromia bungii* late instar larvae.

Insecticide	Effect	Estimate	SE	*t*	*p*
Thiamethoxam	0.01 ppm	−0.013	0.069	−0.18	0.86
	0.1 ppm	6.1 × 10^−16^	0.069	0.00	1.00
	1 ppm	4.8 × 10^−16^	0.069	0.00	1.00
	10 ppm	−0.069	0.069	−1.00	0.33
	100 ppm	0.028	0.069	0.40	0.69
	Week ^1^	7.6 × 10^−17^	0.0081	0.00	1.00
	0.01 ppm:week ^2^	−0.0019	0.011	−0.17	0.87
	0.1 ppm:week	−8.5 × 10^−17^	0.011	0.00	1.00
	1 ppm:week	−6.5 × 10^−17^	0.011	0.00	1.00
	10 ppm:week	−0.053	0.011	−4.64	<0.001
	100 ppm:week	−0.089	0.011	−7.81	<0.001
Dinotefuran	0.01 ppm	−0.035	0.097	−0.36	0.72
	0.1 ppm	0.021	0.097	0.22	0.83
	1 ppm	−0.0063	0.097	−0.066	0.95
	10 ppm	0.13	0.097	1.35	0.19
	100 ppm	−0.014	0.097	−0.14	0.89
	Week	−5.8 × 10^−17^	0.011	0.00	1.00
	0.01 ppm:week	0.0048	0.016	0.30	0.77
	0.1 ppm:week	−0.011	0.016	−0.70	0.49
	1 ppm:week	−9.5 × 10^−4^	0.016	−0.060	0.95
	10 ppm:week	−0.069	0.016	−4.33	<0.001
	100 ppm:week	−0.059	0.016	−3.73	<0.005

^1^ Weeks elapsed after start of insecticide exposure (2, 4, 6, 8, 10, and 12 weeks). ^2^ Interaction between *X* ppm treatment and elapsed weeks.

## Appendix D

**Table A4:** Parameter estimates for GLMs to explain the growth rate of *Aromia bungii* late instar larvae.

Insecticide	Effect	Estimate	SE	*t*	*p*
Thiamethoxam	0.01 ppm	0.0093	0.029	0.33	0.75
	0.1 ppm	0.051	0.029	1.77	0.079
	1 ppm	−0.067	0.029	−2.32	0.021
	10 ppm	−0.27	0.031	−8.55	<0.001
	100 ppm	−0.28	0.031	−9.01	<0.001
	Week ^1^	−0.010	0.0031	−3.36	<0.001
	0.01 ppm:week ^2^	−0.0033	0.0044	−0.75	0.45
	0.1 ppm:week	−0.0065	0.0043	−1.52	0.13
	1 ppm:week	0.0084	0.0043	1.95	0.053
	10 ppm:week	0.0075	0.0069	1.09	0.28
	100 ppm:week	0.023	0.0073	3.19	<0.005
Dinotefuran	0.01 ppm	−0.035	0.030	−1.18	0.24
	0.1 ppm	0.032	0.029	1.10	0.27
	1 ppm	−0.054	0.029	−1.85	0.065
	10 ppm	−0.21	0.030	−6.85	<0.001
	100 ppm	−0.27	0.032	−8.57	<0.001
	Week	−0.010	0.0030	−3.43	<0.001
	0.01 ppm:week	0.0088	0.0043	2.05	<0.05
	0.1 ppm:week	−0.0030	0.0044	−0.70	0.49
	1 ppm:week	0.011	0.0043	2.52	<0.05
	10 ppm:week	0.0049	0.0052	0.94	0.35
	100 ppm:week	0.019	0.0062	3.03	<0.005

^1^ Weeks elapsed after start of insecticide exposure (2, 4, 6, 8, 10, and 12 weeks). ^2^ Interaction between *X* ppm treatment and elapsed weeks.

## Appendix E

**Table A5:** Parameter estimates for GLMMs to explain the level of diet excavation by *Aromia bungii* late instar larvae.

Insecticide	Effect	Estimate	SE	*t*	*p*
Thiamethoxam	0.01 ppm	−0.030	0.17	−0.18	0.86
	0.1 ppm	−0.083	0.17	−0.49	0.62
	1 ppm	−0.092	0.17	−0.55	0.58
	10 ppm	−1.08	0.17	−6.22	<0.001
	100 ppm	−1.47	0.17	−8.56	<0.001
	Week ^1^	−0.081	0.014	−5.66	<0.001
	0.01 ppm:week ^2^	−0.0037	0.021	−0.18	0.86
	0.1 ppm:week	0.034	0.020	1.70	0.091
	1 ppm:week	0.043	0.020	2.12	<0.05
	10 ppm:week	−0.082	0.029	−2.89	<0.005
	100 ppm:week	0.0034	0.027	0.13	0.90
Dinotefuran	0.01 ppm	−0.084	0.18	−0.46	0.65
	0.1 ppm	0.054	0.18	0.30	0.77
	1 ppm	−0.21	0.18	−1.18	0.24
	10 ppm	−0.72	0.18	−3.95	<0.001
	100 ppm	−1.15	0.19	−6.18	<0.001
	Week	−0.081	0.016	−5.02	<0.001
	0.01 ppm:week	0.024	0.023	1.02	0.31
	0.1 ppm:week	0.0048	0.023	0.21	0.84
	1 ppm:week	0.060	0.023	2.59	<0.05
	10 ppm:week	−0.076	0.025	−3.09	<0.005
	100 ppm:week	−0.030	0.028	−1.04	0.30

^1^ Weeks elapsed after start of insecticide exposure (2, 4, 6, 8, 10, and 12 weeks). ^2^ Interaction between *X* ppm treatment and elapsed weeks.
